# Genome sequence of *Ensifer meliloti* strain WSM1022; a highly effective microsymbiont of the model legume *Medicago truncatula* A17

**DOI:** 10.4056/sigs.4608286

**Published:** 2013-12-15

**Authors:** Jason Terpolilli, Yvette Hill, Rui Tian, John Howieson, Lambert Bräu, Lynne Goodwin, James Han, Konstantinos Liolios, Marcel Huntemann, Amrita Pati, Tanja Woyke, Konstantinos Mavromatis, Victor Markowitz, Natalia Ivanova, Nikos Kyrpides, Wayne Reeve

**Affiliations:** 1 Centre for Rhizobium Studies, Murdoch University, Western Australia, Australia; 2 School of Life and Environmental Sciences, Deakin University, Victoria, Australia; 3 Los Alamos National Laboratory, Bioscience Division, Los Alamos, New Mexico, USA; 4 DOE Joint Genome Institute, Walnut Creek, California, USA; 5 Biological Data Management and Technology Center, Lawrence Berkeley National Laboratory, Berkeley, California, USA

**Keywords:** root-nodule bacteria, nitrogen fixation, rhizobia, *Alphaproteobacteria*

## Abstract

*Ensifer meliloti* WSM1022 is an aerobic, motile, Gram-negative, non-spore-forming rod that can exist as a soil saprophyte or as a legume microsymbiont of *Medicago*. WSM1022 was isolated in 1987 from a nodule recovered from the roots of the annual *Medicago orbicularis* growing on the Cyclades Island of Naxos in Greece. WSM1022 is highly effective at fixing nitrogen with *M. truncatula* and other annual species such as *M. tornata* and *M. littoralis* and is also highly effective with the perennial *M*. *sativa* (alfalfa or lucerne). In common with other characterized *E. meliloti* strains, WSM1022 will nodulate but fixes poorly with *M. polymorpha* and *M. sphaerocarpos* and does not nodulate *M. murex*. Here we describe the features of *E. meliloti* WSM1022, together with genome sequence information and its annotation. The 6,649,661 bp high-quality-draft genome is arranged into 121 scaffolds of 125 contigs containing 6,323 protein-coding genes and 75 RNA-only encoding genes, and is one of 100 rhizobial genomes sequenced as part of the DOE Joint Genome Institute 2010 Genomic Encyclopedia for *Bacteria* and *Archaea*-Root Nodule Bacteria (GEBA-RNB) project.

## Introduction

An available source of nitrogen (N) is essential to life on Earth. Although the atmosphere consists of approximately 80% N, the overwhelming proportion of this is present in the form of dinitrogen (N_2_) which is biologically inaccessible to the vast majority of higher organisms. Only a subset of microbes has the necessary molecular machinery to make atmospheric N_2_ bioavailable by enzymatically reducing N_2_ to NH_3_. The fact that plant growth is most commonly limited by the availability of N may have provided the selective pressure for a wide range of plant genera, most of which are legumes, to evolve a symbiotic relationship with these N_2_-fixing microbes. These microsymbionts, collectively termed root nodule bacteria, receive a carbon source from the plant and in return supply the host with biologically fixed N. When these symbiotic interactions are optimally harnessed in agriculture, all the N-requirements of the host can be met, without the need to apply industrially synthesized N-based fertilizers, thereby increasing both the economic and environmental sustainability of the farming system [1].

Forage and fodder legumes play an integral role in sustainable farming practice, providing feed for stock while also enriching soil with bioavailable N. Worldwide, there are approximately 110 million ha of forage and fodder legumes under production [2], of which members of the *Medicago* genus comprise a considerable component. Two bacterial species, *Ensifer meliloti* and *E. medicae* are known to nodulate and fix N_2_ with *Medicago* spp. [3], although they differ in their symbiotic properties on some *Medicago* hosts. Specifically, while *E. medicae* can nodulate and fix N_2_ with *M. murex, M. arabica and M. polymorpha*, *E. meliloti* does not nodulate *M. murex*, does not fix with *M. polymorpha and* fixes N_2_ very poorly with *M. arabica* [4-6].

*E. meliloti* strain WSM1022 was isolated in 1987 from a nodule collected from the annual *M. orbicularis* growing on the Cyclades Island of Naxos in Greece. *E. meliloti* WSM1022 is a highly effective microsymbiont of *Medicago*, forming efficient N_2_-fixing associations with the annual species *M. littoralis* and *M. tornata* [7]. In common with *E. medicae* WSM419 [8], WSM1022 also fixes approximately twice as much N_2_ as *E. meliloti* 1021 on the model legume *M. truncatula* A17 [7]. However, unlike *E. medicae* WSM419, *E. meliloti* WSM1022 is also highly effective with the perennial *M. sativa* (alfalfa or lucerne) [7]. Therefore, *E. meliloti* WSM1022 is a broadly effective microsymbiont of *Medicago* spp. and as such represents a unique tool for the molecular analysis of effective N_2_ fixation with fully sequenced macro-and microsymbionts. Here we present a summary classification and a set of general features for *E. meliloti* strain WSM1022 together with a description of its genome sequence and annotation.

## Classification and features

**Figure 1:**
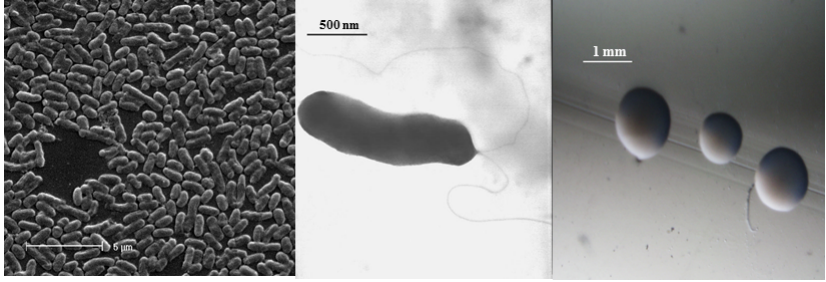
Images of *Ensifer meliloti* WSM1022 using scanning (Left) and transmission (Center) electron microscopy and the appearance of colony morphology on a solid medium (Right).

*E. meliloti* WSM1022 is a motile, Gram-negative rod (Figure 1 Left and Center) in the order *Rhizobiales* of the class *Alphaproteobacteria*. It is fast growing, forming colonies within 3-4 days when grown on half strength Lupin Agar (½LA) [9], tryptone-yeast extract agar (TY) [10] or a modified yeast-mannitol agar (YMA) [11] at 28°C. Colonies on ½LA are white-opaque, slightly domed and moderately mucoid with smooth margins (Figure 1Right).

**Figure 2:**
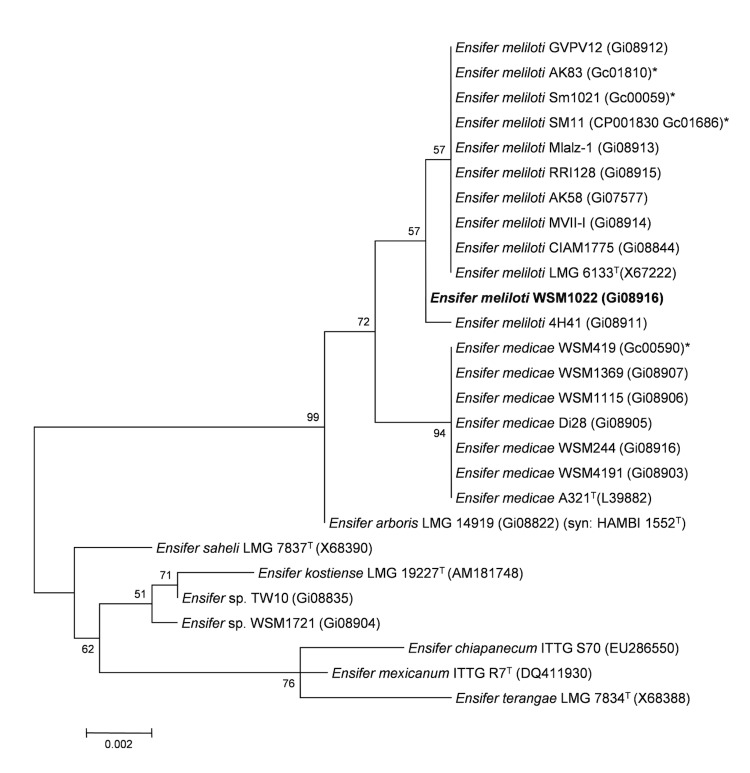
Phylogenetic tree showing the relationship of *Ensifer meliloti* WSM1022 (shown in bold print) to other *Ensifer* spp. in the order *Rhizobiales* based on aligned sequences of the 16S rRNA gene (1,290 bp internal region). All sites were informative and there were no gap-containing sites. Phylogenetic analyses were performed using MEGA, version 5 [25]. The tree was built using the Maximum-Likelihood method with the General Time Reversible model [26]. Bootstrap analysis [27] with 500 replicates was performed to assess the support of the clusters. Type strains are indicated with a superscript T. Brackets after the strain name contain a DNA database accession number and/or a GOLD ID (beginning with the prefix G) for a sequencing project registered in GOLD [28]. Published genomes are indicated with an asterisk.

Minimum Information about the Genome Sequence (MIGS) is provided in Table 1. Figure 2 shows the phylogenetic neighborhood of *E. meliloti* WSM1022 in a 16S rRNA sequence based tree. This strain shares 99.92% and 99.61% sequence identity (over 1290 bp) to the 16S rRNA of the fully sequenced *E. meliloti* 1021 [29] and *E. medicae* WSM419 [8] strains, respectively.

**Table 1:** **Classification and general features of *Ensifer meliloti* WSM1022 according to the MIGS recommendations [**12**]**

**MIGS ID**	**Property**	**Term**	**Evidence code**
	Current classification	Domain *Bacteria*	TAS [13]
		Phylum *Proteobacteria*	TAS [14]
		Class *Alphaproteobacteria*	TAS [15,16]
		Order *Rhizobiales*	TAS [16,17]
		Family *Rhizobiaceae*	TAS [18,19]
		Genus *Ensifer*	TAS [20-22]
		Species *Ensifer meliloti*	TAS [21]
	
	Gram stain	Negative	IDA
	Cell shape	Rod	IDA
	Motility	Motile	IDA
	Sporulation	Non-sporulating	NAS
	Temperature range	Mesophile	NAS
	Optimum temperature	28°C	NAS
	Salinity	Non-halophile	NAS
MIGS-22	Oxygen requirement	Aerobic	TAS [7]
	Carbon source	Varied	NAS
	Energy source	Chemoorganotroph	NAS
MIGS-6	Habitat	Soil, root nodule, on host	TAS [7]
MIGS-15	Biotic relationship	Free living, symbiotic	TAS [7]
MIGS-14	Pathogenicity	Non-pathogenic	NAS
	Biosafety level	1	TAS [23]
	Isolation	Root nodule	TAS [11]
MIGS-4	Geographic location	Naxos, Greece	TAS [11]
MIGS-5	Soil collection date	28 April 1987	IDA
MIGS-4.1	Longitude	37.107772	IDA
MIGS-4.2	Latitude	25.387841	
MIGS-4.3	Depth	0-10cm	
MIGS-4.4	Altitude	Not recorded	

Evidence codes – IDA: Inferred from Direct Assay; TAS: Traceable Author Statement (i.e., a direct report exists in the literature); NAS: Non-traceable Author Statement (i.e., not directly observed for the living, isolated sample, but based on a generally accepted property for the species, or anecdotal evidence). These evidence codes are from the Gene Ontology project [24].

### Symbiotaxonomy

*E. meliloti* strain WSM1022 was isolated in 1987 from a nodule collected from the annual *M. orbicularis* growing on the Cyclades Island of Naxos in Greece. The site of collection was a gentle slope and the soil a sandy-loam texture of pH 7.5-8.0. *E. meliloti* forms nodules (Nod^+^) and fixes N_2_ (Fix^+^) on a range of annual *Medicago* spp. as well as the perennial *M. sativa* (Table 2). In common with other characterized *E. meliloti* strains, WSM1022 does not nodulate *M. murex*, does not fix N_2_ with *M. polymorpha* and *M. arabica* [4,5] and is a poorly effective microsymbiont of *M. sphaerocarpos* [11]. However, WSM1022 is broadly effective with the alkaline soil-adapted annuals *M. littoralis* and *M. tornata* as well as the widely grown perennial forage legume *M. sativa*. In addition, WSM1022 is also a highly effective microsymbiont for the model legume *M. truncatula* A17.

**Table 2:** Nodulation and N_2_ fixation properties of *E. meliloti* WSM1022 on selected *Medicago* spp. Data compiled from [7,11]^†^

**Species Name**	**Cultivar or Accession**	**Growth** **Habit**	**Nodulation**	**N_2_ fixation**	**Comment**
*M. truncatula*	A17	Annual	Nod^+^	Fix^+^	Highly effective
*M. truncatula*	Jemalong	Annual	Nod^+^	Fix^+^	Highly effective
*M. truncatula*	Caliph	Annual	Nod^+^	Fix^+^	Highly effective
*M. littoralis*	Harbinger	Annual	Nod^+^	Fix^+^	Highly effective
*M. tornata*	Tornafield	Annual	Nod^+^	Fix^+^	Highly effective
*M. sphaerocarpos*	Orion	Annual	Nod^+^	Fix^+^	Poorly effective
*M. arabica*	SA36043	Annual	Nod^+^	Fix^-^	No fixation
*M. polymorpha*	Santiago	Annual	Nod^+^	Fix^-^	No fixation
*M. murex*	Zodiac	Annual	Nod^-^	Fix^-^	No nodulation
*M. sativa*	Sceptre	Perennial	Nod^+^	Fix^+^	Highly effective

^†^Note that ‘+’ and ‘-’ denote presence or absence, respectively, of nodulation (Nod) or N_2_ fixation (Fix).

## Genome sequencing and annotation

### Genome project history

This organism was selected for sequencing on the basis of its environmental and agricultural relevance to issues in global carbon cycling, alternative energy production, and biogeochemical importance, and is part of the Community Sequencing Program at the U.S. Department of Energy, Joint Genome Institute (JGI) for projects of relevance to agency missions. The genome project is deposited in the Genomes OnLine Database [28] and an improved-high-quality-draft genome sequence in IMG. Sequencing, finishing and annotation were performed by the JGI. A summary of the project information is shown in Table 3.

**Table 3:** Genome sequencing project information for *E. meliloti* WSM1022.

**MIGS ID**	**Property**	**Term**
MIGS-31	Finishing quality	Improved high-quality draft
MIGS-28	Libraries used	1× Illumina library
MIGS-29	Sequencing platforms	Illumina HiSeq 2000
MIGS-31.2	Sequencing coverage	Illumina: 275×
MIGS-30	Assemblers	Velvet version 1.1.04; Allpaths-LG version r42328
MIGS-32	Gene calling methods	Prodigal 1.4, GenePRIMP
	GOLD ID	Gi08916
	NCBI project ID	78233
	Database: IMG	2510065057
	Project relevance	Symbiotic N_2_ fixation, agriculture

### Growth conditions and DNA isolation

*E. meliloti* WSM1022 was cultured to mid logarithmic phase in 60 ml of TY rich medium [30] on a gyratory shaker at 28°C. DNA was isolated from the cells using a CTAB (Cetyl trimethyl ammonium bromide) bacterial genomic DNA isolation method [31].

### Genome sequencing and assembly

The genome of *Ensifer meliloti* WSM1022 was sequenced at the Joint Genome Institute (JGI) using Illumina technology [32]. An Illumina standard shotgun library was constructed and sequenced using the Illumina HiSeq 2000 platform which generated 12,082,430 reads totaling 1812.4 Mbp.

All general aspects of library construction and sequencing performed at the JGI can be found at the JGI website [31]. All raw Illumina sequence data was passed through DUK, a filtering program developed at JGI, which removes known Illumina sequencing and library preparation artifacts (Mingkun, L., Copeland, A. and Han, J., unpublished). The following steps were then performed for assembly: (1) filtered Illumina reads were assembled using Velvet [33] (version 1.1.04), (2) 1–3 kb simulated paired end reads were created from Velvet contigs using wgsim (https://github.com/lh3/wgsim), (3) Illumina reads were assembled with simulated read pairs using Allpaths–LG [34] (version r42328). Parameters for assembly steps were: 1) Velvet (velveth: 63 –shortPaired and velvetg: –veryclean yes –exportFiltered yes –mincontiglgth 500 –scaffolding no–covcutoff 10) 2) wgsim (–e 0 –1 100 –2 100 –r 0 –R 0 –X 0) 3) Allpaths–LG (PrepareAllpathsInputs:PHRED64=1 PLOIDY=1 FRAGCOVERAGE=125 JUMPCOVERAGE=25 LONGJUMPCOV=50, RunAllpath-sLG: THREADS=8 RUN=stdshredpairs TARGETS=standard VAPIWARNONLY=True OVERWRITE=True). The final draft assembly contained 125 contigs in 121 scaffolds. The total size of the genome is 6.6 Mb and the final assembly is based on 1,812.4 Mbp of Illumina data, which provides an average 275× coverage of the genome.

### Genome annotation

Genes were identified using Prodigal [35] as part of the DOE-JGI annotation pipeline [36]. The predicted CDSs were translated and used to search the National Center for Biotechnology Information (NCBI) nonredundant database, UniProt, TIGRFam, Pfam, PRIAM, KEGG, COG, and InterPro databases. The tRNAScanSE tool [37] was used to find tRNA genes, whereas ribosomal RNA genes were found by searches against models of the ribosomal RNA genes built from SILVA [38]. Other non–coding RNAs such as the RNA components of the protein secretion complex and the RNase P were identified by searching the genome for the corresponding Rfam profiles using INFERNAL (http://infernal.janelia.org). Additional gene prediction analysis and manual functional annotation was performed within the Integrated Microbial Genomes (IMG-ER) platform [39].

## Genome properties

**Figure 3:**
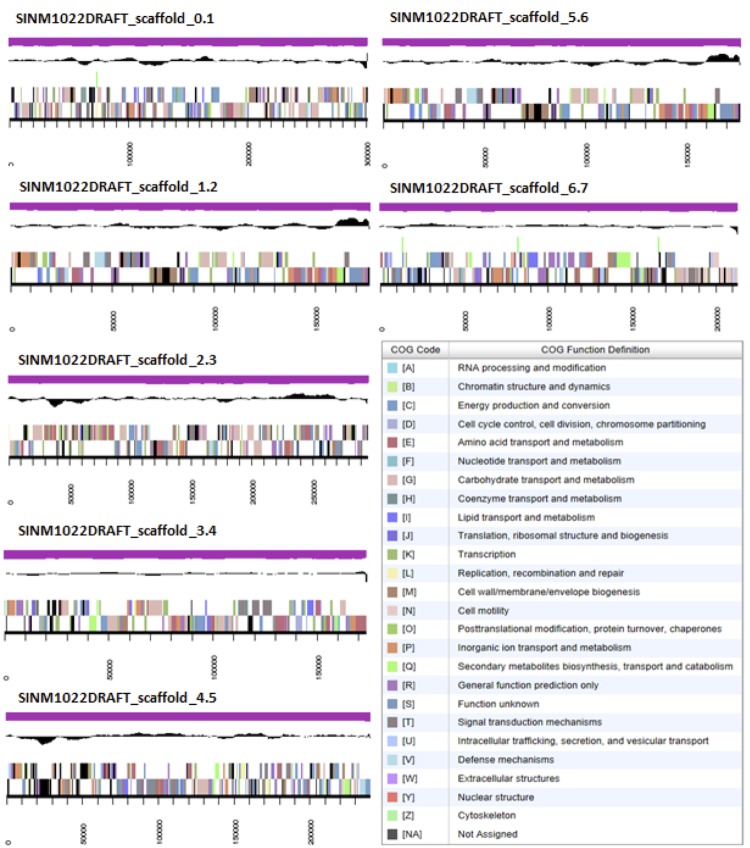
Graphical map of the genome of *Ensifer meliloti* WSM1022 showing the seven largest scaffolds. From bottom to the top of each scaffold: Genes on forward strand (color by COG categories as denoted by the IMG platform), Genes on reverse strand (color by COG categories), RNA genes (tRNAs green, sRNAs red, other RNAs black), GC content, GC skew.

The genome is 6,649,661 nucleotides with 62.16% GC content (Table 4) and comprised of 121 scaffolds (Figure 3) of 125 contigs. From a total of 6,398 genes, 6,323 were protein encoding and 75 RNA only encoding genes. The majority of genes (80.78%) were assigned a putative function whilst the remaining genes were annotated as hypothetical. The distribution of genes into COGs functional categories is presented in Table 5.

**Table 4:** Genome Statistics for *Ensifer meliloti* WSM1022

**Attribute**	**Value**	**% of Total**
Genome size (bp)	6,649,661	100.00
DNA coding region (bp)	5,733,017	86.22
DNA G+C content (bp)	4,133,661	62.16
Number of scaffolds	121	
Number of contigs	125	
Total gene	6,398	100.00
RNA genes	75	1.17
rRNA operons	1	0.02
Protein-coding genes	6,323	98.83
Genes with function prediction	5,168	80.78
Genes assigned to COGs	5,147	80.45
Genes assigned Pfam domains	5,331	83.32
Genes with signal peptides	563	8.80
Genes with transmembrane helices	1,437	22.93
CRISPR repeats	0	

**Table 5:** Number of protein coding genes of *Ensifer meliloti* WSM1022 associated with the general COG functional categories.

**Code**	**Value**	**% age**	**COG Category**
J	194	3.38	Translation, ribosomal structure and biogenesis
A	0	0.00	RNA processing and modification
K	497	8.65	Transcription
L	196	3.41	Replication, recombination and repair
B	1	0.02	Chromatin structure and dynamics
D	38	0.66	Cell cycle control, mitosis and meiosis
Y	0	0.00	Nuclear structure
V	61	1.06	Defence mechanisms
T	235	4.09	Signal transduction mechanisms
M	301	5.24	Cell wall/membrane biogenesis
N	71	1.24	Cell motility
Z	0	0.00	Cytoskeleton
W	1	0.02	Extracellular structures
U	113	1.97	Intracellular trafficking and secretion
O	177	3.08	Posttranslational modification, protein turnover, chaperones
C	357	6.21	Energy production conversion
G	606	10.54	Carbohydrate transport and metabolism
E	623	10.84	Amino acid transport metabolism
F	109	1.90	Nucleotide transport and metabolism
H	200	3.48	Coenzyme transport and metabolism
I	207	3.60	Lipid transport and metabolism
P	312	5.43	Inorganic ion transport and metabolism
Q	158	2.75	Secondary metabolite biosynthesis, transport and catabolism
R	708	12.32	General function prediction only
S	583	10.14	Function unknown
-	1,251	19.55	Not in COGS
Total	5,748	-	-
